# Good maternal and fetal outcomes of three consecutive pregnancies in a Mediterranean woman with Alport syndrome: a case report

**DOI:** 10.1186/s13256-022-03565-7

**Published:** 2022-09-01

**Authors:** Hasan Rawashdeh, Haifaa A. Alchalabi, Ashraf O. Oweis, Haneen Al Jalodi

**Affiliations:** 1 Obstetrics and Gynecology Department, Jordan University of Science and Technology, Irbid, Jordan; 2 Obstetrics and Gynecology Department, Jordan University of Science and Technology (Retired), Irbid, Jordan; 3 Internal Medicine Department, Jordan University of Science and Technology, Irbid, Jordan; 4 Obstetrics and Gynecology Department at King Abdulla University Hospital. Higher specialization in medicine at Jordan University of Science and Technology, Irbid, Jordan

**Keywords:** Alport syndrome, Case report, Fetal outcome, Maternal outcome

## Abstract

**Background:**

Alport syndrome is a rare inherited disorder affecting the glomerular basement membrane, manifested by hematuria and proteinuria that is commonly associated with ocular and hearing defects. There is limited information about the maternal and fetal outcomes of Alport syndrome in pregnancy.

**Case presentation:**

We describe a smooth course of pregnancy, a good maternal outcome, and a good fetal outcome in three consecutive pregnancies for a 35-year-old Mediterranean woman with Alport syndrome over a 10-year duration. Although there was a nephrotic range of progressive proteinuria in all her pregnancies, there was a prompt drop in proteinuria within 2 weeks of her deliveries. She has constantly shown a normal serum creatinine level and a normal serum protein level in all her pregnancies. Apart from a single episode of asymptomatic hypertension in her second pregnancy at 34 weeks of gestation that returned to a normal range immediately after delivery, she was normotensive antenatally and postnatally. She gave birth by cesarean section to three healthy newborns.

**Conclusions:**

A normal prepregnancy creatinine level and a mild range of proteinuria in a patient with normotension, who is not on any medication, are associated with good maternal and fetal outcomes. Furthermore, successful pregnancy that is followed by a normal renal function test might suggest a favorable outcome for any future pregnancy.

## Background

Alport syndrome (AS) is an inherited disorder characterized by progressive glomerulonephritis, deafness, and ocular changes. AS is caused by mutations in any type IV collagen genes *COL4A3*, *COL4A4* (2q36.3 both), and *COL4A5* (Xq22.3). While there are three different genetic forms of Alport syndrome (X-linked, autosomal recessive, and autosomal dominant), it has been suggested that, in females, it is either X-linked in 85% of cases or autosomal recessive in the remaining 15% [1–3].

While chronic renal damage and the need for dialysis are common in males, the disease is milder in females. Renal manifestations of the disease may present merely with hematuria and proteinuria during adolescence in almost all patients. While end-stage renal disease may develop in only 15% by age 60, hearing loss and lenticonus are common by middle age [1, 2].

The exact impact of Alport syndrome on pregnancy is not clear because it is a rare disorder, and the number of reported cases is small. In the literature, the course of pregnancy in women with Alport syndrome has shown variable outcomes, ranging from those with a good outcome characterized by increasing proteinuria that decreases after delivery and a good fetal outcome to the more complicated cases with permanent maternal renal damage and hypertension [4–6].

Furthermore, there are no apparent prognostic factors predicting the outcome of pregnancy in women with Alport syndrome apart from the only study that reported a more favorable outcome among women with normal kidney function and no hypertension prior to pregnancy [7]. There is also insufficient information about the behavior of Alport syndrome in consecutive pregnancies in the same patient.

Hence, we decided to report the good maternal and fetal outcomes of three consecutive pregnancies in a normotensive patient with Alport syndrome who had a normal prepregnancy serum creatinine level and a mild prepregnancy level of proteinuria. Moreover, we suggest that a successful pregnancy that is followed by a normal renal function test might be a good prognostic factor for any future pregnancy.

## Case presentation

A 35-year-old Mediterranean woman was diagnosed with Alport syndrome at the age of 7, when she was investigated for hematuria and proteinuria, confirmed by a renal biopsy on her father, revealing a familial chronic renal disease.

Our patient was descended from a nonconsanguineous parent. Her mother was completely asymptomatic, while her father had been diagnosed with Alport syndrome when he was 42 years of age, and he had been on hemodialysis for the last few years because of chronic renal failure. The pattern of this disease inheritance in her family suggested an X-linked type, as her brothers were asymptomatic. At the same time, her only sister had shown hematuria since she was a teenager. Genetic testing was offered to the patient and her offspring but declined as the test was not insured financially.

After three successful pregnancies and 3 years from her last delivery, the patient is normotensive, had a normal kidney function test, and was not undergoing any specific treatment. However, recently she had been found to have some auditory changes from tinnitus, mild neurosensory hearing loss, and mild lenticular myopia caused by her anterior lenticonus.

### First pregnancy (2009)

At the age of 23, the patient came to the antenatal clinics at 7 weeks of gestation, and an early ultrasound examination confirmed a pregnancy consistent with age. The physical examination was unremarkable, and the blood pressure was 90/70 mmHg. Her baseline investigations showed a hemoglobin level of 9.9 g/dl, 2+ protein from urine analysis, and serum creatinine of 41 µmol/l (62.0–106.0 µmol/l). Twenty-four-hour urine collection measured 1.870 g protein. Combined obstetric and nephrology care was arranged with serial growth assessment, proteinuria, and kidney function testing. She was under strict observation for symptoms and signs of preeclampsia. She was admitted to the obstetric unit for monitoring because of rising proteinuria of 4.300 g in 24 hours and a borderline amniotic fluid index at 34 weeks. Her blood pressure was 100/60 mmHg.

At 37 weeks of gestation, 24-hour urine collection measured 5.694 g protein, creatinine 49 µmol/l, total protein 53.4 g/l (60–80 g/l), and albumin at 27.8 g/l (35–52 g/l). She had a cesarean section at 37 completed weeks for an obstetric reason (breech presentation) and delivered a healthy, 2530 g female newborn baby.

Proteinuria dropped down to the early pregnancy levels shortly after delivery. The patient recovered smoothly after surgery and was discharged home with her newborn infant.

The patient had a follow-up visit at the nephrology clinic after 2 weeks of her puerperium, which revealed proteinuria of +2 and creatinine of 52 µmol/l. The patient did not attend the clinics again until the second pregnancy.

### Second pregnancy (2015)

At age 30, the patient came once more to the antenatal clinics for antenatal care. She was 13 weeks pregnant, confirmed by an early ultrasound examination that revealed a singleton pregnancy consistent with age. The physical examination was unremarkable, and her blood pressure was 95/60 mmHg. There was a +1 protein in the urine, and the creatinine level was 41 µmol/l. Combined obstetric and nephrology care was arranged with serial growth and fetal well-being assessments, as well as the recording of proteinuria and creatinine levels. She was under strict observation for symptoms and signs of preeclampsia. She was admitted at 30 weeks of gestation so that her borderline amniotic fluid index could be assessed. At 34 weeks of gestation, the patient had an elevated blood pressure of 140/89 mmHg, for which she was again admitted to the hospital for observation. Proteinuria was 3.900 g in a 24-hour collection, serum creatinine was 44 µmol/l, hemoglobin level was 9.3 g/dl, and the platelet count was 222 × 10^3^/mm^3^. The total serum protein was 51 g/l, albumin was 27.0 g/l, normal levels of alanine transaminase, and aspartate aminotransferase being 8.9 U/l and 15 U/l, respectively. She had a cesarean section due to a suspected dehiscence scar and gave birth to a male newborn infant weighing 2300 g, who was admitted to the neonatal intensive care unit. The mother’s proteinuria and blood pressure dropped shortly after 2 weeks of her delivery. The mother and the newborn infant were discharged home in good health. The patient did not attend the clinics again until the third pregnancy.

### Third pregnancy (2017)

At age 32, the patient presented to the antenatal clinics for antenatal care. She was 12 weeks pregnant, confirmed by an early ultrasound examination that revealed a singleton pregnancy consistent with age. The physical examination was unremarkable, with a blood pressure of 100/60 mmHg. A 24-hour urine collection showed 1.010 g in 2400 ml. The serum creatinine level was 41 µmol/l. Combined obstetrics and nephrology care was established with serial growth and fetal well-being assessment. At 28 weeks, during a routine antenatal visit, a dipstick for protein showed +3, while the 24-hour urine collection revealed 2.400 g in 1300 ml. Her blood pressure was normal all through her antenatal period. She was under strict observation for symptoms and signs of preeclampsia. At 34 weeks, the protein in the 24-hour urine collection measured 2.900 g in 1200 ml, while serum creatinine was 45 µmol/l and hemoglobin level 9.1 g/dl. A cesarean section was performed at 37 weeks for obstetric reasons, and a male newborn of 2500 g was delivered and sent to the nursery. After 2 days, the mother and her newborn infant were discharged home. After 4 weeks, the patient was reevaluated once more, and she was doing well, with a blood pressure of 110/60 mmHg and a 24-hour urine collection measuring 1.700 g in 2200 ml. There were no auditory or ophthalmological complaints. Late follow-up was lost apart from a few visits documented to the auditory and ophthalmology clinics.

## Discussion and conclusions

Alport syndrome is a rare monogenic disease caused by a mutation in any type IV collagen genes, coding α3, α4, and α5 chains. These collagen chains are present in the basement membranes and Bowman’s capsules in the kidneys, cochlea, retina, lens, cornea, skin, and smooth muscles. The prevalence of this disease is underestimated because many cases of childhood hematuria were diagnosed as being of thin basement membrane or benign familial hematuria. However, on the basis of genetic analysis, many of these cases were reclassified as Alport syndrome, making it the second most common inherited kidney disease after polycystic kidney disease [8]. Consequently, a noticeable rise in the number of pregnant women with Alport syndrome is thus expected in the future. Obstetricians will need to know more about this disease’s course and its pathophysiology in pregnancy.

On reviewing the literature on Alport syndrome in pregnancy and after experiencing three pregnancies in the same patient, we have come to the following observations.

Similar to pregnancies with chronic renal disease, Alport syndrome is generally associated with good maternal outcomes if the prepregnancy creatinine level is normal and the prepregnancy proteinuria is mild in a woman with normotension [7]. The maternal outcome of the three pregnancies described in our report supports this general conclusion in the literature. On the other hand, a persistent postpartum abnormal renal function was described in a patient with a high prepregnancy proteinuria level [9]. Only one case in the literature deviated from this conclusion, where the patient ended up with chronic renal failure after a rapid deterioration of her renal function at 25 weeks of gestation, despite having a normal prepregnancy creatinine level [4]. Therefore, we suggest that the prepregnancy renal function should be integral to the preconception counseling session for the maternal outcome for patients with Alport syndrome. Nevertheless, at the same time, it is vital to make the patient aware that such a conclusion was derived from a limited number of reported cases in the literature.

Progressive proteinuria with advancing gestation is indeed a common finding that was present in all the reported cases of Alport syndrome in the literature and all our patient’s documented pregnancies [10]. There was a prompt regression of proteinuria within 2 weeks of delivery back to prepregnancy levels in the majority of cases documented in the literature. Two sisters had a delayed return to a non-nephrotic range of proteinuria after 6 months of delivery [6], while two other patients developed chronic renal failure after pregnancy and continued to show a nephrotic range of proteinuria [4, 9].

Regarding the fetal outcome, the main risk for the fetus from Alport syndrome is prematurity [7, 10]. In our report, the fetal outcome was considered to be good, apart from the observed amniotic fluid index being at a lower level than normal in the first two pregnancies. It is worth mentioning that, in our case, the body weight of all the newborns delivered was in the lower range of normal. This observation was noticed and recorded [10].

Interestingly, the consistency of the same patient having similar good maternal and fetal outcomes in consecutive pregnancies was also observed in similar case reports [7, 11].

In summary, pregnancy in women with Alport syndrome is certainly a challenge for all parties involved, namely the obstetrician, the nephrologist, and the patient herself. They should be considered high-risk pregnancies and should therefore be monitored carefully for increasing proteinuria, hypertension, and elevated serum creatinine levels, especially at around 34 weeks. Fetal well-being, including the assessment of fetal growth and the amount of liquor, should be checked regularly using gestational age-growth charts. From the patient’s perspective, she believes that outpatient management is a possible, safe, and appropriate way of management.

## Conclusions

We concluded that a normal prepregnancy renal function and a mild range of proteinuria in a patient with normotension who is not on any medication are associated with good maternal and fetal outcomes. Furthermore, successful pregnancy followed by a normal renal function test might suggest a favorable outcome for any future pregnancy.

## Data Availability

The data that support the findings of this study are available from King Abdulla University Hospital, but restrictions apply to the availability of these data, which were used under license for the current study and so are not publicly available. Data are, however, available from the authors upon reasonable request and with permission of King Abdulla University Hospital.
